# Effect of Stocking Density and Biofilm-Based Microalgae on Larvae and Post-Larvae Growth and Settlement Patterns of the Clam *Ruditapes decussatus* (Linnaeus, 1758) in Captivity

**DOI:** 10.3390/ani15243557

**Published:** 2025-12-11

**Authors:** Rania Azirar, Yassine Ouagajjou, Fiz da Costa, Hafsa Janah, Adil Aghzar

**Affiliations:** 1 Research Team of Agricultural and Aquaculture Engineering (G2A), Polydisciplinary Faculty of Larache, Abdelmalek Essaadi University, Tetouan 93000, Morocco; rania.azirar@etu.uae.ac.ma (R.A.); y.ouagajjou@uae.ac.ma (Y.O.); hafsa.janah@etu.uae.ac.ma (H.J.);; 2 Amsa Shellfish Research Station, National Institute of Fisheries Research (INRH), Tetouan 93000, Morocco; 3 AquaCOV, Centro Oceanográfico de Vigo, Instituto Español de Oceanografía (IEO, CSIC), 36390 Vigo, Spain

**Keywords:** *Ruditapes decussatus*, shellfish hatchery, larval rearing, aquaculture, settlement, density, biofilm, Morocco

## Abstract

Larval rearing is a critical phase in shellfish-hatchery production. Thus, optimizing larval survival, growth, and settlement is essential for reliable seed output. This study investigated the effect of rearing density on larvae and settlement performances of the carpet shell clam *Ruditapes decussatus* in captivity. Furthermore, the influence of natural biofilm-based microalgae on larvae settlement and post-larvae performance was also investigated herein. Three larval rearing densities (10, 20, and 40 larvae mL^−1^) were studied during larvae development, and four settlement densities (35, 70, 100, and 140 larvae cm^−2^) were tested during larvae settlement and metamorphosis. The effect of biofilm-based microalgae on larvae settlement and post-larvae pattern was tested at a standard settlement density (70 larvae cm^−2^). High larval density significantly reduced survival (18% at 40 larvae mL^−1^) compared with lower densities (26% and 23% at 20 and 10 larvae mL^−1^, respectively). Significantly higher settlement rate (36% and 33%) and spat yield were observed at 35 and 70 larvae cm^−2^, respectively, compared to high densities (100 and 140 larvae cm^−2^). Remarkably, post-larvae reared under the biofilm treatment showed faster growth performances compared to untreated (control) ones. These findings indicated that lower larval and settlement densities enhance survival and settlement rates. Natural biofilm promoted post-larval growth, and therefore, its use can be an effective strategy to improve *R. decussatus* hatchery outcomes.

## Simple Summary

The carpet shell clam *Ruditapes decussatus* is a valuable species for the blue economy in Morocco and Europe, supporting fisheries, aquaculture, and local livelihoods. However, natural populations are declining due to overfishing and environmental stressors. This study examined the effects of larval and settlement densities on *R. decussatus* survival, growth, and settlement under hatchery conditions and the effect of natural biofilm-based microalgae on settlement and post-larval performance. Lower larval and settlement densities improved survival and settlement rates, while biofilm treatments boosted post-larval growth. These findings offer useful practical strategies for optimizing clam hatchery output, advancing sustainable aquaculture, protecting wild populations, and supporting food security. By improving hatchery management, this work contributes to sustainable development goals and helps reduce pressure on natural clam stocks in both Morocco and Europe.

## 1. Introduction

The grooved carpet shell clam *Ruditapes decussatus* (Linnaeus, 1758) has long supported culturally and economically important fisheries along the Atlantic and Mediterranean coasts and plays a key role in many estuarine ecosystems [1,2,3]. It also holds substantial importance in shellfish production [4]. This species has become a valuable commercial resource in several countries, including Morocco, due to its high demand in both local and international seafood markets [5,6,7]. However, grooved carpet shell populations have faced multiple challenges. Overexploitation driven by high commercial demand has led to significant population decline. Additionally, coastal development and pollution have degraded natural habitats, reducing suitable areas for growth and reproduction, while disease outbreaks have further contributed to the decline of wild populations [8,9,10]. As a result, implementing conservation measures and sustainable management practices is essential to ensure the long-term viability of this important species. In light of this concerning situation, the development of controlled practices, such as hatchery production, is strongly recommended to provide an alternative source of spat to supplement natural recruitment. Controlled production in captivity can help ensure a reliable supply of spat and reduce pressure on wild populations, thereby supporting a more balanced and sustainable approach to meeting both commercial and ecological needs [3,11].

Larval rearing represents a critical and technically demanding stage in bivalve hatchery operations, as it directly influences the viability and productivity of aquaculture systems [11,12]. Successful larval culture requires the maintenance of optimal conditions, including water quality, temperature, nutrition, and stocking density during both broodstock conditioning and larval development [12,13,14]. To maximize production efficiency, hatcheries often employ high larval densities to reduce space requirements and operational costs. However, this practice can lead to zootechnical challenges, such as increased mortality rates and heightened susceptibility to disease outbreaks. Stocking density is a key determinant of larval performance, influencing behavior, dispersal, and competition for food and space in controlled environments [12,13]. Understanding these density-dependent interactions is essential for developing sustainable and environmentally responsible shellfish production strategies. Although density effects have been extensively studied in several bivalve species, there remains a significant knowledge gap regarding the response of *R. decussatus* larvae under hatchery conditions. Species-specific assessments are crucial, as density-related impacts can vary considerably among taxa, and such insights are fundamental for optimizing hatchery protocols for *R. decussatus.*

The early life cycle of most sedentary marine invertebrates includes a planktonic larval phase. At the onset of competence—a physiological state in which larvae acquire the ability to settle and undergo metamorphosis into benthic juveniles—they begin exploring and selecting suitable substrates for attachment [15]. Settlement is strongly mediated by environmental cues, particularly chemical and physical signals associated with biofilm formation, which play a pivotal role in triggering metamorphosis.

In the marine environment, biofilms exist on all surfaces and play a key role in mediating biotic interactions and biogeochemical activities [16]. Additionally, they mediate larval settlement and metamorphosis of many marine invertebrates, and serve as biofilters to absorb and biodegrade excessive nutrients [17,18]. For example, Yu et al. [19] demonstrated that natural biofilm could promote larval settlement of the pearl oyster *Pinctada fucata*, and Campbell et al. [20] showed that biofilms on hard surfaces enhanced larval settlement of the native Eastern oyster *Crassostrea virginica*. Algal biofilms usually enhance settlement [15,21] and facilitate adhesion of invertebrates [22,23]. The settlement intensity may depend on biofilm characteristics [24,25]. The influence of biofilm age on larval settlement is reported to be quantitative since biofilm age is also linked to the densities of bacteria and diatoms [26,27]. Biofilm age could also determine microbial community structure [26] and community metabolic activity [27]. Clams’ settlement, in particular, is positively correlated with biofilm age [26,27]. However, specific properties of biofilms that influence the habitat selection process are not well known [28,29]. Although the effects of biofilms and high larval density on growth and survival have been investigated in other bivalve species, there is still a dearth of species-specific information for *Ruditapes decussatus*. Optimizing hatchery production of this commercially significant clam requires an understanding of these factors. Thus, the objectives of this study are to (i) determine how larval rearing density affects growth and survival, and (ii) determine how biofilm presence affects larval settlement and subsequent growth. Our hypothesis is that (1) higher larval densities may have a detrimental effect on growth and survival, whereas (2) a mature biofilm may improve settlement success and post-settlement growth.

## 2. Materials and Methods

### 2.1. Clam Sampling and Broodstock Conditioning

In February 2022, a total of 135 adult specimens of the local clam *Ruditapes decussatus* (average shell length 24.32 ± 1.51 mm and average live weight of 10.54 ± 1.27 g) were carefully hand-collected from a natural bed population in Dakhla Bay (Southern Atlantic coast of Morocco: 3°43′ N 15°57′ W). The collected clams were conditioned for nine weeks under a controlled temperature of 22.0 ± 1.0 °C and physicochemical parameters such as salinity (35 ppt), pH (8.03 ± 0.02), and dissolved oxygen (5.10 ± 0.40 mg L^−1^). The clams were fed with a constant diet of microalgae at a ratio of 3% of their dry meat weight, and their gonadal maturity was checked once a week. For each evaluation, five clams were randomly selected and examined microscopically to observe gametogenic development. Subsequently, their Condition Index (CI) and Gonadal Condition Index (GCI) were calculated to determine the best moment to induce spawning. The microalgae strains used during broodstock conditioning were *Isochrysis galbana* (CCAP 927/1), *Chaetoceros calcitrans* (CCMP1315), and *Nannochloropsis gaditana* (CCAP 849/5), which were cultivated at the Amsa Shellfish Station of the INRH (Morocco) under controlled laboratory conditions.

### 2.2. Spawning and Fertilization

Thermal shock stimulation was used to induce clams to spawn following nine weeks of conditioning. Three alternating cycles of cold seawater (10.0 ± 0.5 °C) and warm seawater (28.1 ± 1.0 °C) were applied to the clams. Each clam, male or female, was put in a small container with 200 milliliters of filtered seawater to continue releasing gametes (either sperm or oocytes) once they had begun. After that, 120 sperm were used to fertilize each oocyte. Fertilized eggs were collected, rinsed with 0.2 µm-filtered and UV-irradiated seawater, and then incubated in a 1000 L circular polycarbonate tank under static water conditions without circulation at a temperature of 22.0 ± 1.0 °C using a thermo-stat.

### 2.3. Larval Rearing and Post-Larvae Settlement

After 48 h, D-shaped larvae were collected using a 20 µm mesh screen and were reared in 20 L tanks. Total veliger numbers were estimated by counting three aliquots under a profile projector. Early D-larvae were distributed into three initial densities (10, 20, and 40 larvae mL^−1^; D10, D20, and D40, respectively, in triplicate). Larval growth and survival were monitored every two days over 16 days.

At the pediveliger stage (day 16), cultures were gently homogenized for 1–2 min, sieved (150 µm mesh), cleaned in UV-treated water, and resuspended in 1 L of filtered water. The number of pediveligers was determined by counting three 25 µL subsamples under a binocular microscope, and total pediveligers (*Nt*) were calculated as follows:

(1) Nt=Np×Vr

where *Np* is the number of pediveligers per mL in the subsample, and *Vr* is the total suspension volume.

For settlement density treatments, the required number of pediveligers per cylinder (surface area S = 700 cm^2^) was calculated, and the corresponding suspension volume (*Vt*) was determined using the following:

(2) Vt=(N1×V1)/Nt

Settlement rate (*Sr*) was calculated as follows:

(3) $$Sr\left(\%\right)=(\frac{Nf}{Ni})\times 100$$

where *Nf* is the number of post-larvae settled, and *Ni* is the number of seeded pediveligers. Final post-larvae were weighed to determine total numbers (*Nf* = *Wt*/*Wi*), where *Wt* is the total post-larvae weight, and *Wi* is the average individual weight calculated from subsamples (*Wi* = *Wss*/*Nss*). Post-larval production per unit area was calculated as follows:

(4) $$\mathrm{Pr}\left(post-larvae\;{\mathrm{cm}}^{2}\right)=Nf/S$$

### 2.4. Algal Biofilms and Post-Larvae Settlement

#### 2.4.1. Preparation of Algal Biofilms and Settlement Rearing

Biofilms were prepared 15 days before the experiment by immersing clean meshes (total surface portions 700 cm^2^) in a mixture of microalgae (*I. galbana*, *C. calcitrans*, and *T. suecica*). The control treatment used sterile, clean meshes and was kept free of biofilm by not being exposed to any algal inoculation.

For the initial inoculation of the biofilm treatment, equal amounts of each microalgae species were combined for a total cell density of 3 × 10^2^ cells mL^−1^ per species. The meshes were kept under static conditions in filtered, UV-treated seawater while immersed in the algal suspension. A flow-through system was not used, instead, the water was gently aerated to avoid sedimentation. To sustain algal growth, the suspension was renewed every five days. After 15 days, visual inspection confirmed that biofilms covered more than 80% of the mesh surface, forming a thick layer.

Following the preparation of algal biofilms, the settlement system was equipped. Six replicates of the biofilm treatment and six replicates of the control were used. Post-larvae were distributed at an approximate initial density of 50,000 larvae per screen (70 larvae cm^−2^). Subsequently, feeding was added, and aeration was initiated, using micro-filtered water treated with UV, with a temperature ranging between 19.1 ± 1.0 °C and 21.1 ± 1.0 °C.

At the end of the larval rearing process, the presence of *Vibrio* spp. was detected by bacteriological analysis of water and larval samples. Samples were cultured on thiosulfate-citrate-bile salts-sucrose (TCBS) agar for *Vibrio* spp. detection. Only the surviving larvae were selected and transferred to the settlement system, and the presence of *Vibrio* spp. did not appear to influence larval survival outcomes.

#### 2.4.2. Spat Grading

Following 11 weeks of settlement, size grading (large and small) of individuals in each treatment was performed to prevent competition for space and nutrients and to allow smaller individuals to continue growing in favorable conditions. A 0.5 mm mesh screen was used for size separation. The collected individuals were placed in small containers before being redistributed onto the screens to estimate their width, length, weight, and total number.

### 2.5. Feeding Process

During the early larval rearing process, a mixture of *I. galbana* (60%), *C. calcitrans* (35%), and *T. suecica* (5%) was supplied daily to rearing tanks, and the water was renewed every 2 days. Every 48 h, samples were taken from each density to estimate survival and mean shell length. In the early larval stage (from day 2 to 8 post-fertilization, pf), only *I. galbana* and *C. calcitrans* were provided as food. From day 9 pf onwards (late larvae phase), *T. suecica* was introduced, with a food composition of *I. galbana* (50%), *C. calcitrans* (40%), and *T. suecica* (10%). During the settlement process, a mixture of the three microalgae was used to feed the post-larvae after metamorphosis with an average quantity of 100–150 cell/µL (Table 1).

### 2.6. Statistical Analyses of Data

All data were first checked for normality and homogeneity of variance. Percentage data (survival and settlement rate) were arcsine square-root-transformed prior to analysis to meet assumptions of ANOVA [30].

The effect of larval stocking density on growth (shell length, in µm) and survival rate (%) was analyzed using one-way ANOVA. Following significant ANOVA results, Tukey’s Honest Significant Difference (HSD) test was applied as a post hoc test for pairwise comparisons among treatment groups.

Similarly, settlement rate (%) and spat yield (spat cm^−2^) were analyzed with one-way ANOVA, followed by Tukey HSD post hoc comparisons to identify differences between densities. Statistical significance was accepted at *p* < 0.05, and highly significant differences were reported at *p* < 0.01 and *p* < 0.001.

Additionally, to assess the relationship between larval rearing density and final shell length at day 14, a linear regression analysis was performed. The coefficient of determination (R^2^) and *p*-values were calculated to evaluate the significance of the correlation.

## 3. Results

### 3.1. Effect of Stocking Density on Larval Rearing

**Figure 1:**
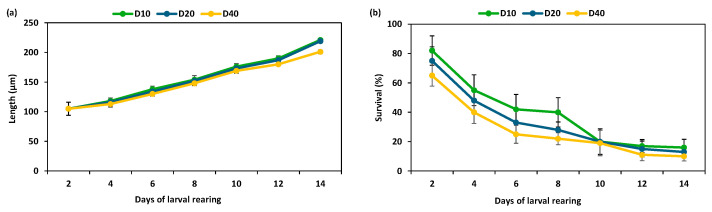
Growth (µm) and survival rate (%) of larvae according to different stocking densities: D10, D20, and D40 (larvae mL^−1^). (**a**): effect of stocking density on growth in length (µm) during larval rearing; (**b**): effect of density on survival rate (%) during clams’ larvae rearing.

The performance of the larvae under varying stocking densities (10, 20, and 40 larvae mL^−1^) is illustrated in Figure 1 in terms of final shell length (µm) on day 14 and survival rate (%). The larvae reared at the lowest density (D10) reached the highest shell length on day 14 (>200 µm), whereas those reared at D20 and D40 showed similar but lower final shell lengths. Larval growth was strongly negatively correlated with rearing density (R^2^ > 0.98), indicating that higher densities resulted in reduced shell length. Survival rates declined as density increased: larvae at D10 and D20 had the highest survival (≈23% and 26%, respectively), while survival at D40 was considerably lower (≈18%). No *Vibrio* spp. had an influence on larval survival or settlement outcomes.

Larvae reared at the highest density (D40) showed significantly reduced growth (200.65 ± 2.92 µm), while D10 and D20 had the highest mean shell length (220.81 ± 2.39 µm and 217.48 ± 3.19 µm, respectively). There was a statistically significant difference in the length and survival rate at the different rearing densities (one-way ANOVA, F = 18.47, *p* < 0.05; F = 13.67, *p* < 0.05), respectively (Table 2). Overall, these results show that larval stocking density has a statistically significant influence on survival and growth.

Pairwise comparisons of survival among density treatments (D10, D20, and D40) revealed that only high D40 and low D10 densities revealed significant difference in terms of survival and growth (F = 10.65, *p =* 0.037, and F = 11.765, *p =* 0.041), whereas all other pairwise comparisons for both survival and length were not significant (Table 3).

### 3.2. Effect of Stocking Density on Settlement

**Figure 2:**
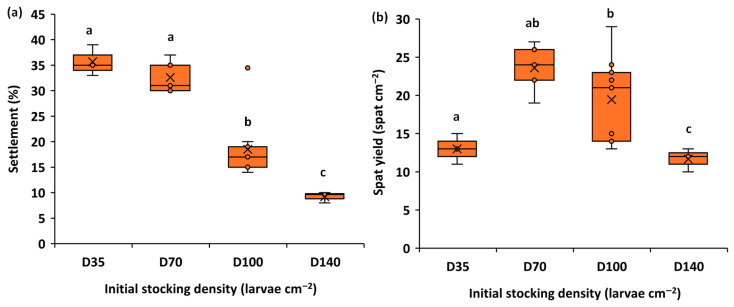
Settlement rate (%) (**a**) and spat yield (spat cm^−2^) (**b**) of *Ruditapes decussatus* under different initial stocking densities (D35, D70, D100, and D140). Boxplots with different letters indicate statistically significant differences among treatments (Tukey HSD, *p* < 0.05).

According to Figure 2, larval density significantly impacts settlement efficiency and final spat yield. The highest settlement rates were observed at the lowest larval densities: D35 and D70, reaching 36% and 33%, respectively. Conversely, the highest densities, D100 and D140, resulted in substantially lower settlement rates of 19% and 9%. However, with a high settlement rate at D35, the low initial larval number resulted in a relatively low spat yield (13 spat cm^−2^). Similarly, the highest density, D140, produced the lowest spat yield (9 spat cm^−2^) due to the very low settlement rate. In contrast, optimal production was achieved at moderate densities. D70 and D100 provided the best balance, yielding both substantial settlement rates and the highest overall spat yields (24 and 19 spat cm^−2^, respectively). These results indicate that intermediate stocking densities are optimal for maximizing final production by balancing the initial larval number with settlement efficiency.

According to statistical analysis (Table 4), larval density had a highly significant influence on settlement rate (F = 14.88, *p* < 0.001) and spat yield (F = 13.09, *p* < 0.001).

Settlement varied considerably between D140 and the other density treatments. Pairwise comparisons showed that settlement at D140 was significantly lower than at D35 (14.671 *), D70 (14.892 *), and D100 (13.015 *) (Table 5).

### 3.3. Effect of Biofilm on Post-Settlement Performance

#### 3.3.1. Effect of Biofilms on Post-Larvae Performance

**Figure 3:**
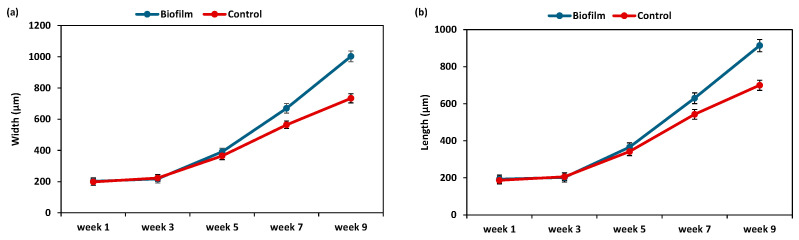
Growth variation (µm) in post-larvae under biofilm and control conditions during settlement. (**a**): post-larvae width variation and (**b**): post-larvae length variation.

During the early post-settlement period, growth trends (length and width) were comparable across treatments during the first weeks (1st to 3rd week), indicating no important differences. Post-larvae in the biofilm treatment, however, showed a steeper growth trend during the late settlement period, with a sharp increase after week 6. By the 9th week, the average shell length of the biofilm treatment was almost 1003 µm and 914 µm (shell width and length, respectively), while the control remained below 734 µm and 700 µm (shell length and shell width, respectively) (Figure 3).

Results of statistical analysis showed that biofilm treatment did not significantly affect either width or length during the early settlement phase (F = 1.675 and F = 1.653, *p* > 0.1) for width and length, respectively. During the late settlement phase, significant effects were revealed for length (F = 12.41 *, *p* = 2.1 × 10^−2^) and width (F = 14.24 *, *p* = 3.7 × 10^−2^) (Table 6).

#### 3.3.2. Effect of Biofilms on Spat Size

##### Effect on Spat Shell Length Classes

**Figure 4:**
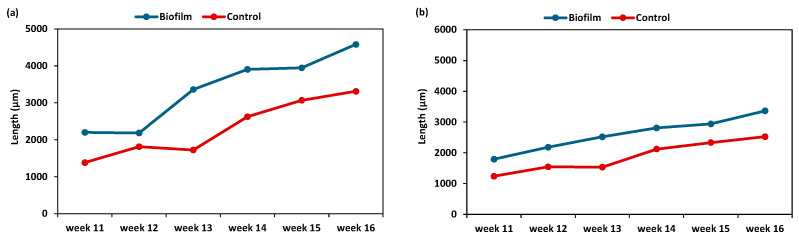
Length variation (µm) of head and tail batch clam spat under biofilm and control conditions during settlement. (**a**): head batch and (**b**): tail batch.

Two post-larvae shell length batches were obtained after grading. According to the findings of Figure 4, post-larvae growth under biofilm-based microalgae treatment showed a higher shell length (head = 4579 ± 34 µm and tail = 3363 ± 12 µm) than the control group for both batches (head = 3311 ± 26 µm and tail = 2524 ± 10 µm) during the duration of the experiment.

##### Shell Length Classes

**Figure 5:**
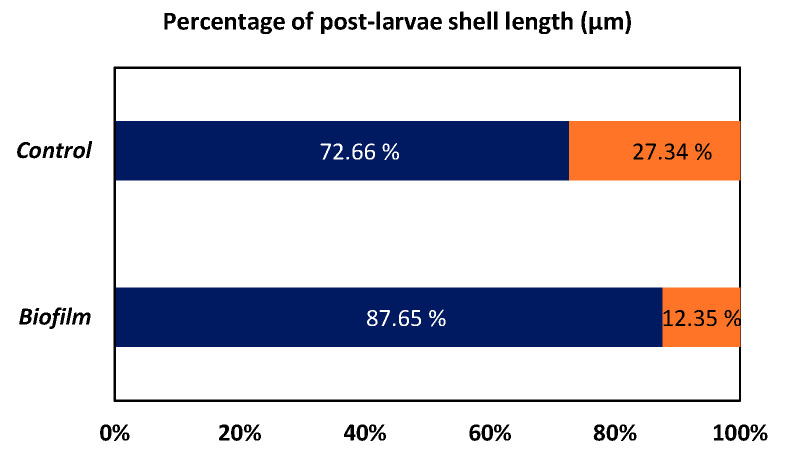
Percentage of post-larvae shell length batches (head, blue; tail, orange).

The biofilm showed a higher percentage of head batch (87.7%) than the control (72.7%). By contrast, biofilm treatment revealed less tail batch percentage (12.4%) compared to the control (27.4%) (Figure 5).

## 4. Discussion

Stocking density is a key factor influencing the success of larval and post-larval performance during rearing in captivity [12,13]. In the present study, the effect of stocking density on the survival and growth of *Ruditapes decussatus* larvae was systematically investigated to understand both larval and post-larval responses. Bivalve mollusks at larval stages are sensitive to high stocking density, which negatively affects growth performance and survivorship in the studied species, such as *Meretrix meretrix* [31], *Ruditapes philippinarum* [32], and *Mulinia edulis* [33].

Our results demonstrated a clear negative correlation between larval survival and stocking density, with mortality rates increasing as density increased. These findings are consistent with Doroudi et al. [34], who reported that moderate densities (20–25 larvae mL^−1^) yielded the highest survival rates for the oyster *Pinctada margaritifera* larvae, suggesting that overly high densities impose physiological stress that reduces survival. High larval densities have consistently been shown to reduce survival and overall larval quality, primarily due to limited food availability, spatial competition, oxygen depletion, and cumulative environmental stressors [12,13,35]. Under hatchery conditions, where food availability may be limited in batch culture after several hours, food scarcity can result in lower growth and survival, and developmental abnormalities [36,37,38]. Competition for space and oxygen deficiency not only slows growth but can also trigger mass mortalities under extreme conditions [39,40]. Additional environmental stressors, such as fluctuations in temperature and salinity, further compound these challenges, directly impacting larval health and viability [41,42].

In this study, stocking density also significantly influenced larval growth patterns. After 16 days, larvae reared at higher densities displayed the lowest growth, whereas shell length gain was higher at low densities. According to earlier research [12,43,44], optimal space and food availability at lower densities promote quicker growth and healthier development, which is consistent with these findings. Higher densities, on the other hand, lead to competition for scarce resources, which slows growth and lowers survival [12,45]. In *M. meretrix* larvae at the veliger stage, an increase in density from 5 larvae to 60 larvae mL^−1^ resulted in reduced growth and survival rate [31]. Therefore, careful control of larval densities is necessary for effective rearing in order to guarantee enough space and food, which improves larval survival, growth, and health.

Interestingly, our results on post-larval settlement reveal a trade-off between settlement rate and spat yield. While higher initial settlement rates were observed at low stocking densities, the moderate density (D70) ultimately yielded the highest spat yield. This suggests that although more larvae initially settle at very low densities, post-settlement survival and growth are optimized at moderate densities where competition is reduced but not minimal, highlighting a balance between settlement success and post-settlement performance [12,39]. These findings align with observations in other bivalve species, such as *M. meretrix*, *M. edulis* [31,33], and *R. philippinarum* [32].

Larval settlement is strongly influenced by environmental cues, particularly microalgal biofilms. Biofilms provide both chemical and structural cues that facilitate attachment and metamorphosis. Research on *M. edulis* and *Crassostrea ariakensis* has demonstrated that the composition and maturity of biofilms significantly affect settlement success [27,46]. Our study revealed that microalgal biofilms, specifically those composed of *I. galbana*, *C. calcitrans*, and *T. suecica*, significantly enhance the settlement and growth of *R. decussatus* larvae. The biofilm may act as a nutritional resource, providing microalgae and other nutrients, while simultaneously serving as a settlement cue that triggers earlier metamorphosis, resulting in a longer growth period for post-larvae [25,26,47,48].

The post-larvae reared with a biofilm showed greater growth in both width and length compared to the control group. This enhanced growth is likely attributable to the biofilm’s components, which include essential microalgae, nutrients, and immune-stimulating substances [25,48]. Specifically, the specific microalgae used (*I. galbana*, *C. calcitrans*, and *T. suecica*) may act as both a direct food source and a stimulant for feeding behavior, enhancing growth performance [26,27]. The progressive maturation of the biofilm likely amplified these effects over time, as the density and diversity of microalgae increased, improving nutrient availability and release of growth-promoting compounds. Similar positive results concerning growth have been observed in other bivalve species, such as *M. galloprovincialis* [15].

The observed enhancement of post-larval growth by biofilms during the later settlement period (after week 5) may be attributed to biofilm maturation. As the biofilm ages, its increased density and microalgal diversity likely improve nutrient availability, release growth-promoting compounds, and enhance feeding stimulation for settling larvae [25,26,27]. Additionally, mature biofilms may provide stronger chemical settlement cues that trigger earlier or more synchronized metamorphosis, allowing larvae to colonize the biofilm more efficiently and extend their growth period [46,47]. These combined factors likely explain why growth differences between biofilm and control groups became more pronounced during the later stages of settlement.

The presence of microalgae biofilm not only enhanced overall growth but also influenced the size distribution of clam spat between the head and tail batches. The head batch, represented by the bigger spat, exhibited significantly larger shell length and width compared to the tail batch (small spat). This difference can be attributed to the earlier onset of feeding and biofilm grazing by the head batch spat, allowing them to benefit from a longer feeding period and better access to the biofilm matrix. Similar growth heterogeneity has been reported in other bivalve species, where the head batch showed higher growth than the tail ones [39]. As reported by Albentosa et al. [49] and Pérez-Camacho et al. [50], the availability and quality of algal resources are key factors in determining growth in bivalve spat. Consequently, the head batch individuals settled earlier and may use the most active and nutrient-rich stage of the biofilm, whereas the tail batch encountered a partially degraded biofilm with reduced microbial activity and nutrient content.

Furthermore, this size difference may have been strengthened by the competition between spats for biofilm resources. Because the head batch spat grew more quickly, they might have monopolized food and space, causing mechanical interference and shading that reduced the tail batch individuals’ ability to feed. In bivalve seed culture, early settlers frequently exhibit superior growth performance and survival, a phenomenon known as dominance-driven growth heterogeneity [39]. Because early settlers were able to fully benefit from the nutritional and biochemical advantages of the biofilm, the biofilm’s beneficial role in encouraging early settlement consequently intensified size differentiation in an indirect manner.

When these observations are combined, it becomes clear that hatchery success depends on striking a balance between stocking density and suitable environmental cues. While the growth and management of biofilms encourage settlement and fixation, low-to-moderate stocking densities lessen competition, enhancing larval growth and survival. By maximizing these variables, it is possible to greatly increase the number of healthy juveniles produced in captivity, boost aquaculture productivity, and aid restoration initiatives for bivalves, including *Ruditapes decussatus*.

## 5. Conclusions

In summary, to maximize larval growth, survival, and post-settlement performance, hatchery optimization requires a careful balance between stocking density, food availability, and environmental management. Moderate densities, such as 10–20 larvae mL^−1^ during larval rearing, promote higher growth and survival, making them suitable for large-scale production. During settlement, moderate densities around 70 larvae cm^−2^ yielded the highest spat production, successfully balancing competition and post-settlement survival. Biofilms are equally crucial as they not only provide essential settlement cues but also serve as a vital source of nutrients for newly settled spat. Early settlers benefit from the most active and nutrient-rich biofilm stages, a factor that significantly enhances growth and survival, particularly during the later settlement period. Therefore, a comprehensive understanding of larval density, food supply, and biofilm dynamics is essential to optimize hatchery practices. Adopting recommended density ranges and biofilm-management strategies can enhance spat yield, reduce mortality, and increase the cost-effectiveness of large-scale *Ruditapes decussatus* production in hatcheries.

## Figures and Tables

**Table 1:** Feeding schedule and microalgal cell densities applied at different developmental stages of *Ruditapes decussatus* during hatchery rearing.

Larval Stage/Feeding	Microalgae Species	Percentage (by Cell Count)	Cell Density (Cells µL^−1^)
Early larvae (Day 2–8)	*I. galbana*	≈55%	20, gradually increased to 50
	*C. calcitrans*	≈45%	25–30, gradually increased to 60
Late larvae (Day 9–16)	*I. galbana*	50%	50–75
	*C. calcitrans*	40%	50–75
	*T. suecica*	10%	10–15
Post-larvae (settlement)	*I. galbana*, *C. calcitrans*, *T. suecica*	100% (mixture)	100–150

**Table 2:** One-way analysis of variance (one-way ANOVA) of the effect of stocking density on length and survival after 14 days of larval culture. Df (degree of freedom), F (Fisher test), * (significant at *p* < 0.05).

Initial Density (larvae mL^−1^)	Length (µm)					Survival (Normalized Values)				
	Mean +/− SD	Df Factor	Df Residual	F	*p*-Value	Mean +/− SD	Df Factor	Df Residual	F	*p*-Value
D10	220.81 ± 2.39	2	6	18.47	2.4 × 10^−2^ *	15.76 ± 2.19	2	6	13.67	3.2 × 10^−2^ *
D20	217.48 ± 3.19					12.83 ± 2.28				
D40	200.65 ± 2.92					9.59 ± 1.82				

**Table 3:** Pairwise comparisons of larvae survival and growth (shell length) between densities D10, D20, and D40 (*F* value). ^ns^ (not significant) and * (significant at *p* < 0.05).

	Density	D10	D20	D40
Survival	D10	-	0.088 ^ns^	0.037 *
	D20		-	0.073 ^ns^
	D40			-
Length	D10	-	0.074 ^ns^	0.041 *
	D20		-	0.065 ^ns^
	D40			-

**Table 4:** Statistical analysis ANOVA of the effect of stocking density on settlement rate and spat yield of post-larvae of the clam (*R. decussatus*). Di (initial density larvae cm^−2^), SD (standard deviation), Df (degree of freedom), F (Fisher test), and *** *p* < 0.001.

Di	Settlement Rate (%)				Spat Yield (Spat.cm^−2^)			
	Mean +/− SD	Df (Factor; Residual)	F	*p*-Value	Mean +/− SD	Df (Factor; Residual)	F	*p*-Value
D35	35.85 ± 10.23	3; 8	14.88	2.4 × 10^−4^ ***	13.23 ± 2.65	3; 8	13.09	3.1 × 10^−4^ ***
D70	33.35 ± 7.35				23.82 ± 5.25			
D100	17.77 ± 1.67				19.04 ± 1.79			
D140	9.38 ± 5.26				9.29 ± 3.42			

**Table 5:** Pairwise comparisons of settlement spat yield between groups D35, D70, D100, and D140. ^ns^ (not significant) and * (significant at *p* < 0.05).

	Density	D35	D70	D100	D140
Settlement (%)	D35	-	0.091 ^ns^	0.034 *	0.024 *
	D70		-	0.041 *	0.021 *
	D100			-	0.038 *
	D140				-
Spat yield	D35	-	0.035 *	0.041 *	0.086 ^ns^
	D70		-	0.072 ^ns^	0.032 *
	D100			-	0.037 *
	D140				-

**Table 6:** Statistical analysis ANOVA of the effect of biofilm on spat growth of the clam (*R. decussatus*). F (Fisher test), ^ns^ (not significant), * (significant at *p* < 0.05), and ** *p* < 0.01.

	Week 1	Week 3	Week 5	Week 7	Week 9
Width	F = 1.675 ^ns^	F = 1.453 ^ns^	F = 17.34 *	F = 14.24 **	F = 13.28 **
Length	F = 1.653 ^ns^	F = 1.458 ^ns^	F = 15.43 *	F = 12.41 **	F = 12.98 **

## Data Availability

The original data presented in the study are openly available in FigShare at https://doi.org/10.6084/m9.figshare.30674681.v1. The dataset is freely available to use with appropriate citation under Creative Commons Attribution 4.0 International Public License.
